# Proteomic and Functional Analyses of the Virion Transmembrane Proteome of Cyprinid Herpesvirus 3

**DOI:** 10.1128/JVI.01209-17

**Published:** 2017-10-13

**Authors:** Catherine Vancsok, M. Michelle D. Peñaranda, V. Stalin Raj, Baptiste Leroy, Joanna Jazowiecka-Rakus, Maxime Boutier, Yuan Gao, Gavin S. Wilkie, Nicolás M. Suárez, Ruddy Wattiez, Laurent Gillet, Andrew J. Davison, Alain F. C. Vanderplasschen

**Affiliations:** a Immunology-Vaccinology, Department of Infectious and Parasitic Diseases, Fundamental and Applied Research for Animals and Health, Faculty of Veterinary Medicine, University of Liège, Liège, Belgium; b Indian Institute of Science Education and Research Thiruvananthapuram, CET Campus, Thiruvananthapuram, India; c Proteomic and Microbiology, Research Institute of Biosciences, University of Mons, Mons, Belgium; d Maria Sklodowska-Curie Institute, Oncology Center, Gliwice Branch, Gliwice, Poland; e MRC-University of Glasgow Centre for Virus Research, Glasgow, United Kingdom; Northwestern University

**Keywords:** cyprinid herpesvirus 3, alloherpesvirus, herpesvirus, proteome

## Abstract

Virion transmembrane proteins (VTPs) mediate key functions in the herpesvirus infectious cycle. Cyprinid herpesvirus 3 (CyHV-3) is the archetype of fish alloherpesviruses. The present study was devoted to CyHV-3 VTPs. Using mass spectrometry approaches, we identified 16 VTPs of the CyHV-3 FL strain. Mutagenesis experiments demonstrated that eight of these proteins are essential for viral growth *in vitro* (open reading frame 32 [ORF32], ORF59, ORF81, ORF83, ORF99, ORF106, ORF115, and ORF131), and eight are nonessential (ORF25, ORF64, ORF65, ORF108, ORF132, ORF136, ORF148, and ORF149). Among the nonessential proteins, deletion of ORF25, ORF132, ORF136, ORF148, or ORF149 affects viral replication *in vitro*, and deletion of ORF25, ORF64, ORF108, ORF132, or ORF149 impacts plaque size. Lack of ORF148 or ORF25 causes attenuation *in vivo* to a minor or major extent, respectively. The safety and efficacy of a virus lacking ORF25 were compared to those of a previously described vaccine candidate deleted for ORF56 and ORF57 (Δ56-57). Using quantitative PCR, we demonstrated that the ORF25 deleted virus infects fish through skin infection and then spreads to internal organs as reported previously for the wild-type parental virus and the Δ56-57 virus. However, compared to the parental wild-type virus, the replication of the ORF25-deleted virus was reduced in intensity and duration to levels similar to those observed for the Δ56-57 virus. Vaccination of fish with a virus lacking ORF25 was safe but had low efficacy at the doses tested. This characterization of the virion transmembrane proteome of CyHV-3 provides a firm basis for further research on alloherpesvirus VTPs.

**IMPORTANCE** Virion transmembrane proteins play key roles in the biology of herpesviruses. Cyprinid herpesvirus 3 (CyHV-3) is the archetype of fish alloherpesviruses and the causative agent of major economic losses in common and koi carp worldwide. In this study of the virion transmembrane proteome of CyHV-3, the major findings were: (i) the FL strain encodes 16 virion transmembrane proteins; (ii) eight of these proteins are essential for viral growth *in vitro*; (iii) seven of the nonessential proteins affect viral growth *in vitro*, and two affect virulence *in vivo*; and (iv) a mutant lacking ORF25 is highly attenuated but induces moderate immune protection. This study represents a major breakthrough in understanding the biology of CyHV-3 and will contribute to the development of prophylactic methods. It also provides a firm basis for the further research on alloherpesvirus virion transmembrane proteins.

## INTRODUCTION

Cyprinid herpesvirus 3 (CyHV-3; species Cyprinid herpesvirus 3, genus Cyprinivirus, family Alloherpesviridae, order Herpesvirales), also known as koi herpesvirus (KHV), is the etiological agent of a lethal disease in common and koi carp. The common carp (Cyprinus carpio) is one of the main fish grown for human consumption (1). Moreover, its colorful ornamental varieties (koi carp) represent one of the most lucrative markets for individual freshwater fish (2, 3). As a result, CyHV-3 is considered to be the archetypal fish herpesvirus and is the subject of a growing number of studies (4).

The CyHV-3 genome is 295 kbp in size and thus the largest described among all herpesviruses (5). It encodes 155 potential protein-coding open reading frames (ORFs), a large number of which lack similarity to other herpesvirus genes (5–7). Nonetheless, CyHV-3 virions present the characteristic herpesvirus morphology (8), consisting of an icosahedral capsid containing the genome surrounded by an amorphous layer of proteins termed the tegument and enveloped in a lipid membrane bearing virion transmembrane proteins (VTPs) (8).

The protein composition of CyHV-3 virions has been investigated by mass spectrometry-based proteomic approaches for one European and two Chinese strains (9, 10). These analyses led to the identification of 46 virion proteins, of which 34 were detected in all three strains. Among these 34 proteins, 13 were classified as VTPs (9, 10). Since none of these proteins are related to viral proteins with known roles, it is not possible to predict their functions.

VTPs mediate key functions in the herpesvirus infectious cycle, such as binding virions to the cell and mediating entry, providing immune evasion mechanisms, facilitating virion morphogenesis, and promoting virion egress from the host cell. The expression of VTPs on the virion surface may also make them targets for neutralizing antibodies. As a result, it is important to study VTPs in order to understand the biology of infection and to aid in the design of candidate vaccines (attenuated, nonreplicative, or subunit). However, current knowledge of CyHV-3 VTPs is very limited. For example, it is not known whether any of the VTPs identified to date are required for viral replication *in vitro*.

Here, we performed proteomic and functional analyses of CyHV-3 VTPs. The proteome of CyHV-3 virions was revisited. Based on this new information and that published previously (9, 10), 16 VTPs were identified and selected for further study. Using recombination technologies, VTPs that are essential to viral growth *in vitro* were identified. Furthermore, the effects of deleting nonessential VTPs on viral growth *in vitro* and virulence *in vivo* were investigated. The knowledge gained represents a major breakthrough in understanding the biology of CyHV-3 and provides a firm basis for further research on alloherpesvirus VTPs.

## RESULTS AND DISCUSSION

### Viral protein composition of CyHV-3 virions.

**FIG 1:**
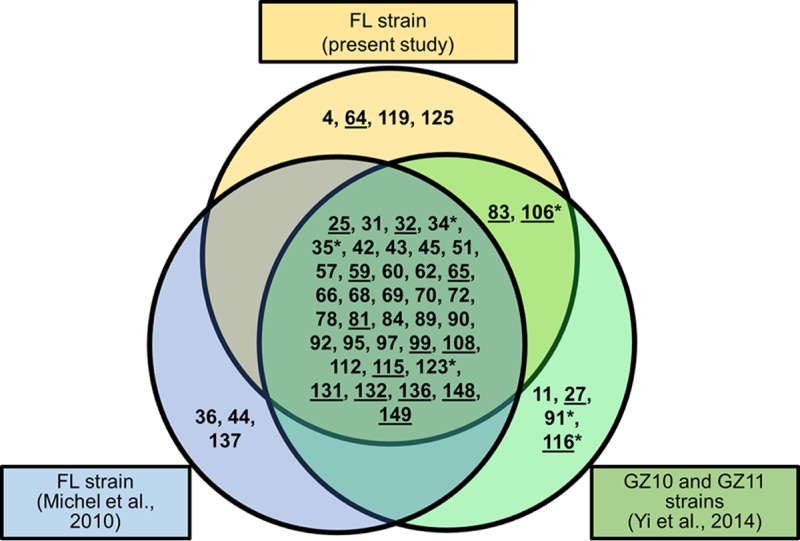
CyHV-3 virion proteome. Schematic representation of CyHV-3 virion-associated proteins identified in independent studies: upper circle, analyses of the European FL strain performed in the present study; lower left circle, analyses of the FL strain performed in a former study (9); and lower right circle, analyses of two Chinese strains (GZ10 and GZ11) (10). Numbers represent CyHV-3 ORFs. Asterisks indicate viral proteins that were detected in only one of the two Chinese isolates. Predicted transmembrane proteins are underlined.

We took advantage of the experience gained during our previous studies of the virion proteomes of herpesviruses (9, 11–13) to revisit the protein composition of extracellular CyHV-3 virions reported by us and others (9, 10). We selected a strategy based on SDS-PAGE separation of proteins, followed by liquid chromatography-tandem mass spectrometry (LC-MS/MS) of tryptic peptides, because this approach has been shown to enhance the recovery of peptides derived from proteins that are prone to aggregation or contain hydrophobic domains (9, 11–13). The data listed in Table 1 represent independent analyses of three independent preparations of CyHV-3 virions. A total of 43 viral proteins were identified (based on detection in at least two of the preparations; Table 1 and Fig. 1). This number is identical to that determined by Yi et al. (10). Comparison of the proteomes derived from both studies revealed 39 proteins in common, with four proteins in each study that were not identified in the other (Fig. 1).

**TABLE 1:**
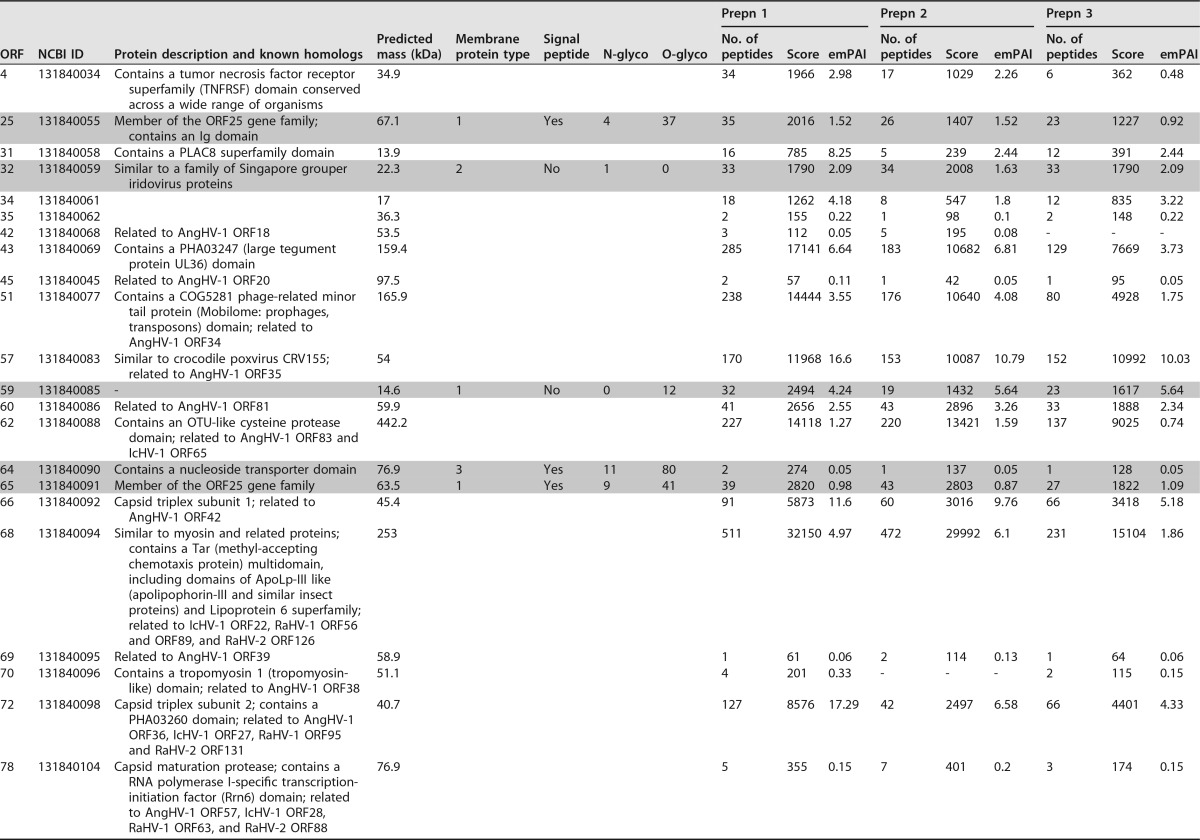

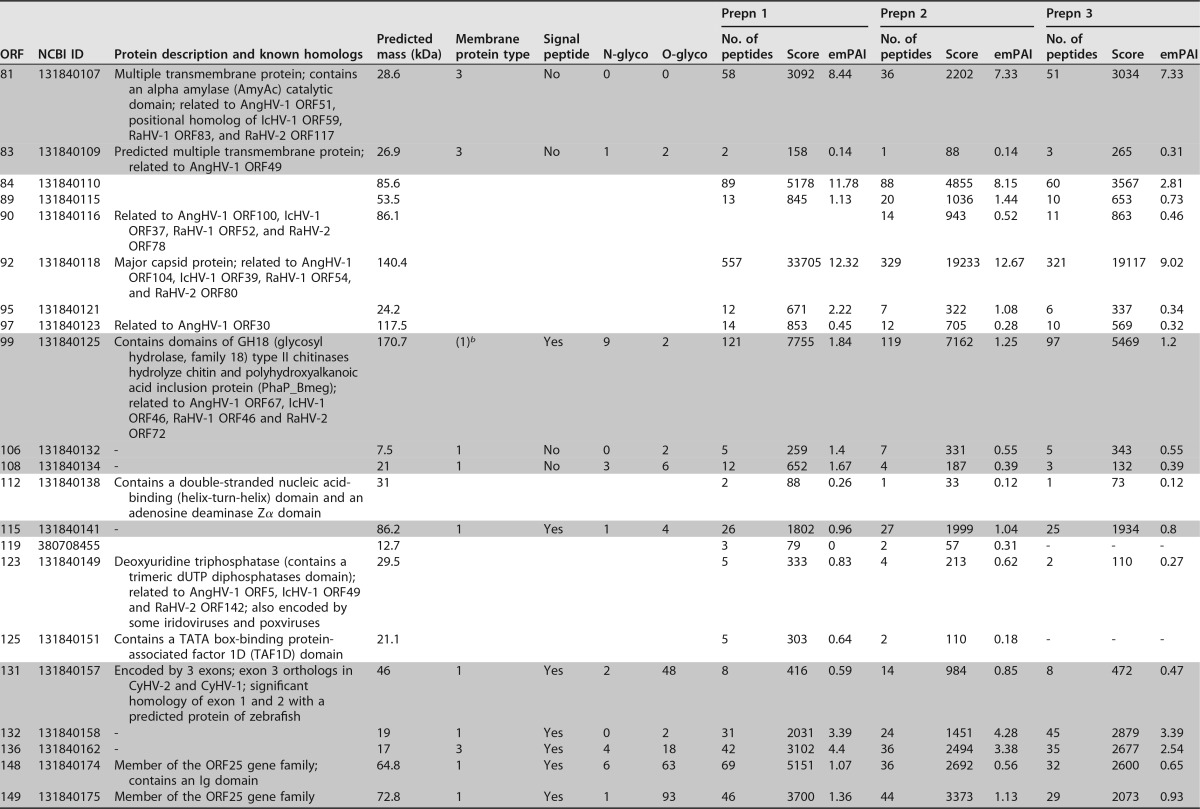
Proteins identified by 2D LC-MS/MS in purified CyHV-3 virions^*a*^

a Data are restricted to CyHV-3 proteins detected by SDS-PAGE and LC-MS/MS in at least two of the three independent preparations of purified virions. Predicted transmembrane proteins are shaded in gray. Protein features were based on previous publications (6, 9) or predictions made using TMHMM, SignalP, NetNGlyc 1.0, and the NetOGlyc 3.1 server from the CBS web site. Similarity and domain analyses were performed by BLASTP N-glyco and O-glyco, number of N glycosylations and O glycosylations, respectively.

^*b*^ No transmembrane domain was detected by software prediction. This sample was classified as a putative type 1 transmembrane protein on the basis of significant similarity to previously studied viral membrane proteins.

Yi et al. (10) identified ORF11, ORF27, ORF91, and ORF116 as potential virion proteins, whereas we did not. The lack of detection of ORF27 in our study was anticipated because this coding region is disrupted in the FL strain, as has been reported for other CyHV-3 strains (5). ORF91 and ORF116 were not detected in any of the preparations in the present study, and were identified by Yi et al. (10) for only one of the two Chinese strains, with detection being based on a single peptide that was supported by a low Mascot score (10). Similarly, ORF11 was detected on the basis of only one or two peptides (depending on the isolate) with relatively low Mascot scores. Among the four proteins that were detected in the present study and not by Yi et al. (10), two were detected in all three replicates (ORF4 and ORF64), and the two other were detected in only two replicates (ORF119 and ORF125). Finally, three proteins identified in our previous study were detected neither by Yi et al. (10) nor in the present study. Taking into account that these proteins were detected initially at relatively low abundances (9), it is likely that they represented contamination by nonstructural proteins.

In the context of the published bioinformatic analyses of the CyHV-3 genome (5, 6), the results presented above suggest that the FL strain of CyHV-3 encodes 16 predicted VTPs: ORF25, ORF32, ORF59, ORF64, ORF65, ORF81, ORF83, ORF99, ORF106, ORF108, ORF115, ORF131, ORF132, ORF136, ORF148, and ORF149.

### CyHV-3 VTPs essential to viral growth *in vitro*.

**FIG 2:**
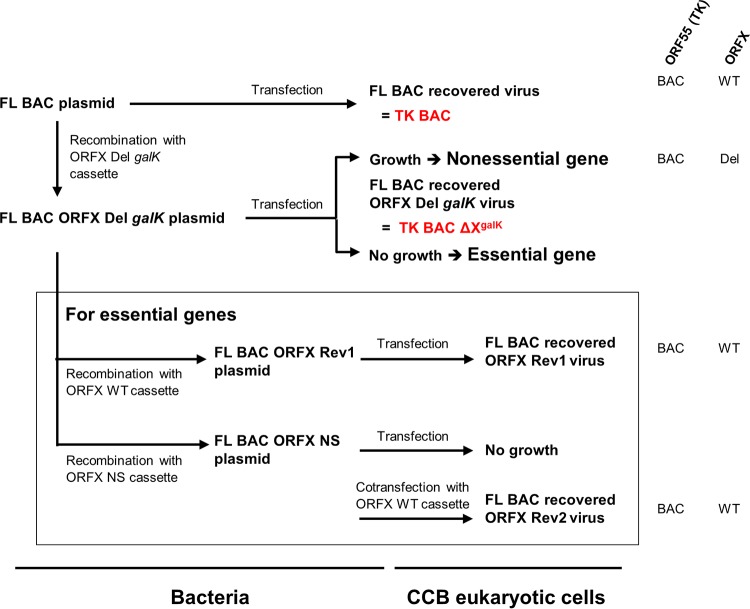
Production of CyHV-3 recombinants to identify essential and nonessential VTPs. Deleted recombinant plasmids were produced for each ORF predicted to encode a VTP (FL BAC ORFX Del *galK* plasmids, with “X” standing for the number of the ORFs tested; these included ORF25, ORF32, ORF59, ORF64, ORF65, ORF81, ORF83, ORF99, ORF106, ORF108, ORF115, ORF131, ORF132, ORF136, ORF148, and ORF149). The effect of the deletion on the ability of the BAC plasmid to reconstitute infectious virus was tested by transfection into permissive CCB cells. Subsequently, for each gene identified as essential, two additional recombinant plasmids were produced to revert to wild-type ORFX sequence (FL BAC ORFX Rev1) or to insert a nonsense mutation (FL BAC ORFX NS). Plasmids were transfected into CCB cells to determine their ability to induce reconstitution of infectious virus. As an additional revertant control, FL BAC ORFX NS plasmids were cotransfected into CCB cells together with a fragment encoding the WT sequence of ORFX and flanking regions, in order to facilitate reversion to wild-type ORFX sequence by recombination in eukaryotic cells (FL BAC ORFX Rev2). To simplify the reading of the manuscript, some recombinants were given a short name (in red). The right part of the Fig. summarizes the genotype of the strains for ORF55 (TK) and ORFX. Del, deleted; WT, wild type; BAC, presence of the BAC cassette in the 3′ end of ORF55.

**FIG 3:**
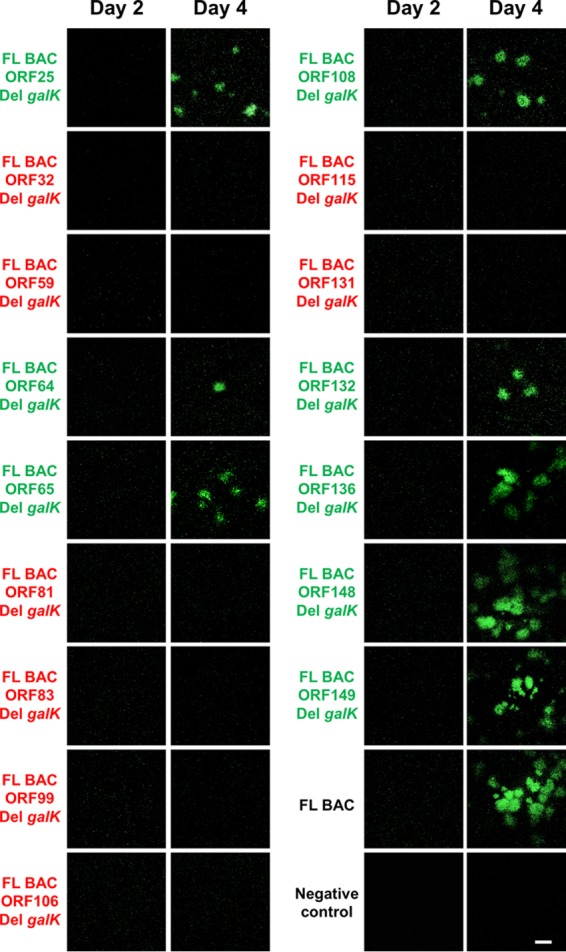
Effect of deleting genes encoding VTPs on the ability of CyHV-3 FL BAC recombinant plasmids to reconstitute infectious virus. CCB cells were transfected with the plasmids indicated. At 2 and 4 dpi, the cells were examined by epifluorescence microscopy (the BAC cassette encodes EGFP). Scale bar, 1 mm. Plasmids able or not able to reconstitute infectious virus are labeled in green or red, respectively.

The roles of CyHV-3 VTPs in viral replication *in vitro* were investigated by using bacterial artificial chromosome (BAC) cloning and prokaryotic recombination technologies to generate viral mutants (Fig. 2). To facilitate the reconstitution of infectious viruses from recombinant plasmids, the BAC cassette was left in the viral genome leading to viruses expressing a truncated form of thymidine kinase (TK; encoded by ORF55) and enhanced green fluorescent protein (EGFP) (the BAC cassette is inserted at the 3′ end of ORF55 and encodes an EGFP expression cassette). The effect of TK truncation on viral replication *in vitro* and virulence *in vivo* has been documented previously. TK truncation was shown to have no effect on viral growth *in vitro* and to reduce virulence slightly *in vivo* (14). For each ORF predicted to encode a VTP, a single-gene-deletion recombinant plasmid was produced by replacing the ORF by a *galK* expression cassette. The molecular structures of all recombinant BAC plasmids produced were confirmed by a combined SacI digestion and Southern blotting approach and by sequencing the regions used to target recombination (data not shown). We then investigated the ability of the recombinant plasmids to reconstitute infectious virus after transfection into permissive CCB cells (Fig. 3). Transfection was monitored by detecting EGFP-expressing cells (since the BAC cassette encodes an EGFP reporter gene) at 2 days postinfection (dpi). Examination of cell cultures at 4 dpi revealed the formation of viral plaques for the FL BAC plasmid (used as a positive control) and the BAC plasmids with ORF25, ORF64, ORF65, ORF108, ORF132, ORF136, ORF148, or ORF149 deleted, thereby demonstrating that these ORFs are nonessential for viral growth *in vitro*. Reconstituted viruses (i.e., viruses mutated in nonessential genes) were amplified, and their genomes were validated by full-length genome sequencing (data not shown) prior to further investigations (see below).

**FIG 4:**
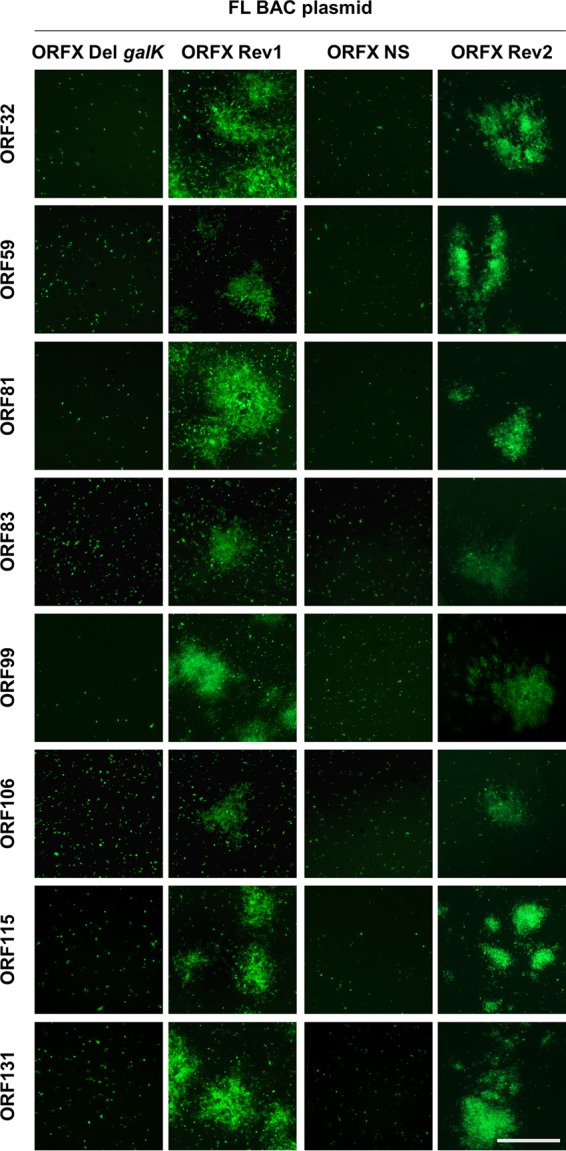
Testing plasmids recombinant for CyHV-3 ORFs predicted to encode essential VTPs. For ORFs identified as essential in Fig. 3, additional recombinant plasmids (ORFX Rev1 and ORFX NS) were produced (see Fig. 2, description in the rectangular frame) and tested for their ability to reconstitute infectious virus after transfection into CCB permissive cells. At 6 days posttransfection, the cells were examined by epifluorescence microscopy for detection of EGFP. Scale bar, 1.5 mm.

Transfection of BAC plasmids with ORF32, ORF59, ORF81, ORF83, ORF99, ORF106, ORF115, or ORF131 deleted did not induce the formation of CyHV-3 plaques (Fig. 3), suggesting that these ORFs encode VTPs essential for viral growth *in vitro*. To test this hypothesis further, additional recombinant BAC plasmids were produced (Fig. 2). The molecular structures of all recombinant BAC plasmids described below were confirmed by a combined SacI digestion and Southern blotting approach and by sequencing the regions used to target recombination (data not shown). To exclude the possibility that the lack of infectivity of the deleted recombinant BAC plasmids resulted from unexpected mutations generated during BAC manipulation, plasmids encoding revertant wild-type (WT) VTP ORF were derived (FL BAC ORFX Rev1 plasmids, Fig. 2) from the deleted BAC plasmids. Transfection of each of the revertant plasmids into CCB cells led to viral growth (Fig. 4, second column). To exclude the possibility that the lack of infectivity observed for the deleted BAC plasmids resulted from a polar effect of the deletion on the expression of a nearby essential gene, a recombinant plasmid in which the ORF was disrupted by a nonsense (NS) mutation was derived for each putative essential VTP gene (FL BAC ORFX NS plasmids, Fig. 2). Transfection of these plasmids into CCB cells did not induce plaque formation (Fig. 4, third column). To exclude the possibility that the lack of infectivity of the NS recombinant BAC plasmids resulted from unexpected mutations generated during BAC manipulation at the stage of NS mutagenesis, the FL BAC ORFX NS plasmids were transfected into CCB cells, together with a WT DNA fragment containing the relevant ORF and flanking regions, in order to induce reversion to a WT sequence after homologous recombination in eukaryotic cells. All plasmids led to viral growth when cotransfected with WT DNA fragments (Fig. 4, fourth column). In summary, the results demonstrated that ORF32, ORF59, ORF81, ORF83, ORF99, ORF106, ORF115, and ORF131 encode VTPs essential to viral growth *in vitro*.

These experiments identified essential *versus* nonessential VTPs of the FL strain of CyHV-3, as summarized in Table 2. To test whether the findings extend across the species Cyprinid herpesvirus 3, the ORFs encoding VTPs were sequenced from nine CyHV-3 strains with different geographical origins and passage histories (Table 2). All of the ORFs encoding essential VTPs were intact and exhibited a limited number of polymorphisms. In contrast, various ORFs encoding nonessential VTPs were truncated by disruptive mutations (highlighted in red in Table 2), the precise pattern depending on the strain.

**TABLE 2:** Polymorphisms observed among CyHV-3 strains in ORFs encoding VTPs

Gene	Requirement in FL strain	Mutation^*a*^
ORF32	Essential	No mutation
ORF59	Essential	No mutation
ORF81	Essential	No mutation
ORF83	Essential	Several aa inserted in M3, T, and J
ORF99	Essential	Several aa changed in KHV-I, M3, T, and J
ORF106	Essential	No mutation
ORF115	Essential	One aa changed in T
ORF131	Essential	One aa changed in KHV-I, FL, Cavoy, and T
ORF25	Nonessential	Several aa inserted in M3, T, J, and GZ11
ORF27	Nonessential	**Frameshifted in U, KHV-I, T, and GZ11; large regions deleted in FL; stop codon insertion in the beginning of the ORF in Cavoy**; several aa changed in M3 and J
ORF64	Nonessential	**Frameshifted** and several amino acids deleted or inserted **in M3, T, and J**
ORF65	Nonessential	One aa deleted in KHV-I, FL, Cavoy, I, and E; one aa inserted and several aa deleted in M3, T, and J; one aa changed in GZ11
ORF108	Nonessential	One aa inserted, one aa changed and **a stop codon inserted at the end of the ORF**, several aa lost **in M3, T, and J**
ORF132	Nonessential	One aa changed in KHV-I
ORF136	Nonessential	Several aa inserted and **a stop codon inserted in the middle of the ORF in M3, T, and J**
ORF148	Nonessential	Several aa inserted in KHV-I, Cavoy, and GZ11; Several aa deleted in M3, T, and J
ORF149	Nonessential	One aa changed in KHV-I, Cavoy, and E, 13 aa inserted in M3 and J

a Boldfacing indicates mutations that create an in-frame stop codon or frameshift. aa, amino acid(s).

### Effects of deleting genes encoding nonessential VTPs on CyHV-3 growth *in vitro*.

Considerations of the CyHV-3 gene arrangement suggested that deletion of ORF25, ORF64, ORF65, ORF108, ORF132, ORF136, or ORF148 would be unlikely to affect the expression of neighboring genes. In contrast, deletion of ORF149 might affect the expression of ORF148, resulting in the phenotype of TK BAC Δ149^galK^ representing the absence of expression of both proteins. To test this hypothesis, TK BAC Δ148^galK^ and TK BAC Δ149^galK^ plaques were stained with monospecific polyclonal antibodies (pAbs) against ORF148 or ORF149. Staining of TK BAC Δ149^galK^ plaques with the anti-ORF148 antibodies demonstrated the expression of the ORF148 protein (data not shown), implying that TK BAC Δ149^galK^ is adequate for investigating the effects of deleting ORF149.

**FIG 5:**
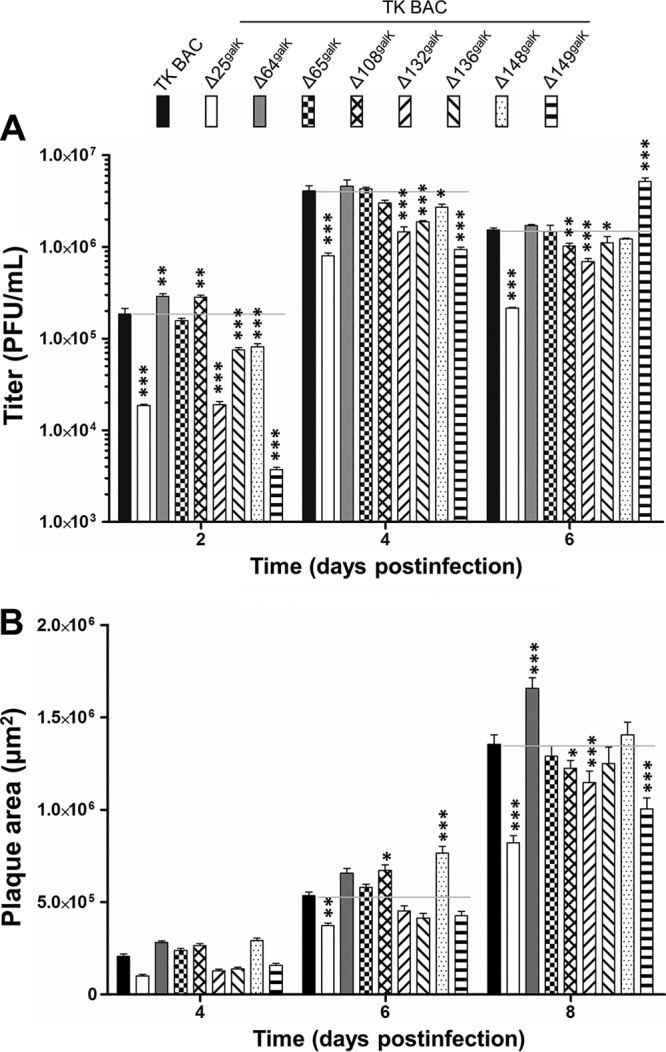
Effects of deleting nonessential VTPs on CyHV-3 growth *in vitro*. (A) Multistep growth curves. CCB cells were infected with the viruses indicated and the titer (PFU/ml) in the cell supernatant was determined at the indicated time points postinfection. Data presented are the means ± the standard errors of the mean (SEM) of triplicate measurements. Significant differences between the deleted viruses and the TK BAC virus were tested by using two-way ANOVA, taking genotype and time postinfection as variables. (B) Plaque size assay. CCB cells were infected with the viruses indicated, and plaque areas were measured over time. The data presented are means ± the SEM for the measurement of 25 randomly selected plaques. Two-way ANOVA was used to test the significance of the results, taking genotype, time postinfection, and interaction between genotype and time postinfection as variables (*, *P* < 0.05; **, *P* < 0.01; ***, *P* < 0.001).

The effect of deleting genes encoding nonessential VTPs on viral growth *in vitro* was investigated by multistep growth assay (Fig. 5A) and plaque size assay (Fig. 5B). Deletion of ORF25, ORF132, ORF136, ORF148, or ORF149 impaired CyHV-3 replication in the multistep growth assays to various degrees (based on observation of a significant effect for at least two consecutive time points). The more pronounced effect was observed for deletion of ORF25, which was associated with a 10-fold reduction in viral titer. In contrast, deleting ORF64, ORF65, or ORF108 had no major effect on CyHV-3 growth. The plaque size assays (Fig. 5B), based on the results collected at 8 dpi, revealed that deleting ORF25, ORF108, ORF132, or ORF149 resulted in reduced plaque size, whereas deleting ORF64 led to an increased plaque size. Deleting ORF65, ORF136, or ORF148 did not significantly affect plaque size.

### Effect of deleting genes encoding nonessential VTPs on CyHV-3 virulence *in vivo*.

**FIG 6:**
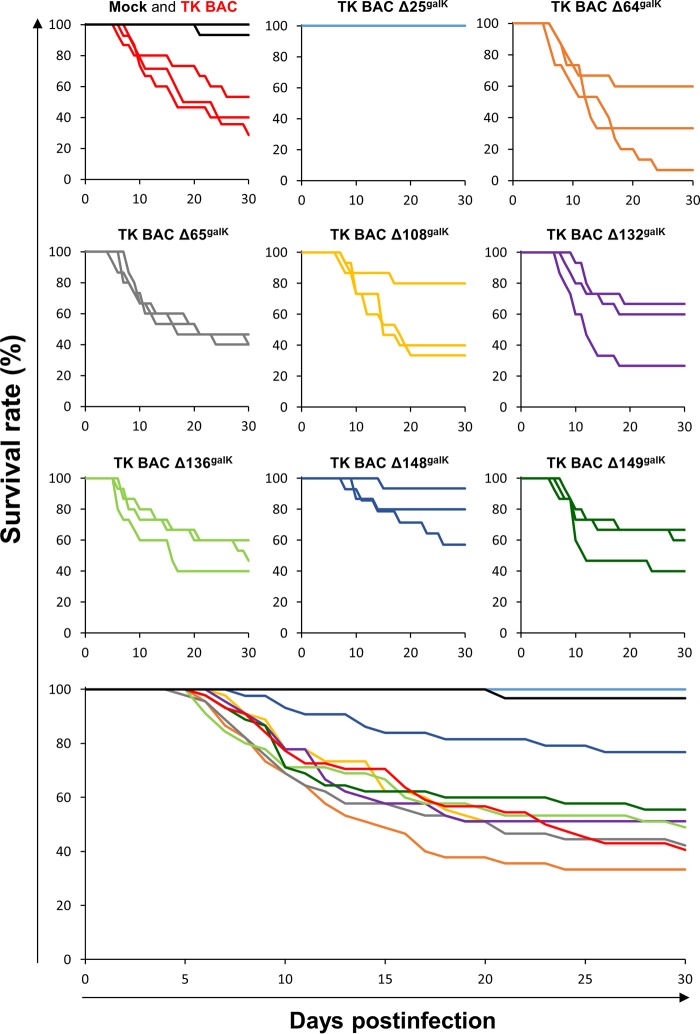
Effect of deleting genes encoding nonessential VTPs on CyHV-3 virulence. The virulence of the indicated recombinant viruses was tested in carp (triplicate groups each consisting of 15 subjects; average weight, 1.90 ± 0.68 g, and average age, 3 months old). On day 0, fish were infected for 2 h by immersion in water containing 800 PFU/ml or mock infected. Survival rate was measured over a period of 30 days. The nine upper panels show the survival curves observed for replicates. The lower panel shows the mean survival curves based on the three replicates.

All recombinant viruses deleted for a nonessential ORF (TK BAC ΔX^galK^, where X is the number of the ORF) were tested in triplicate for their virulence in carp (Fig. 6). Fish infected with the TK BAC virus (used as a positive control) exhibited all the clinical signs associated with CyHV-3 disease, including apathy, folding of the dorsal fin, hyperemia, increased mucus secretion, skin lesions, suffocation, erratic swimming, and loss of equilibrium. At 30 dpi, the mean survival rate of TK BAC-infected fish was 41%. Fish inoculated with TK BAC Δ64^galK^, TK BAC Δ65^galK^, TK BAC Δ108^galK^, TK BAC Δ132^galK^, TK BAC Δ136^galK^, or TK BAC Δ149^galK^ exhibited comparable effects, leading to similar survival rates at 30 dpi (ranging between 33 and 56%). Conversely, fish infected with TK BAC Δ148^galK^ exhibited mild disease and a higher survival rate (77%, *P* < 0.001). Fish inoculated with TK BAC Δ25^galK^ expressed no symptoms, and all survived the infection.

**FIG 7:**
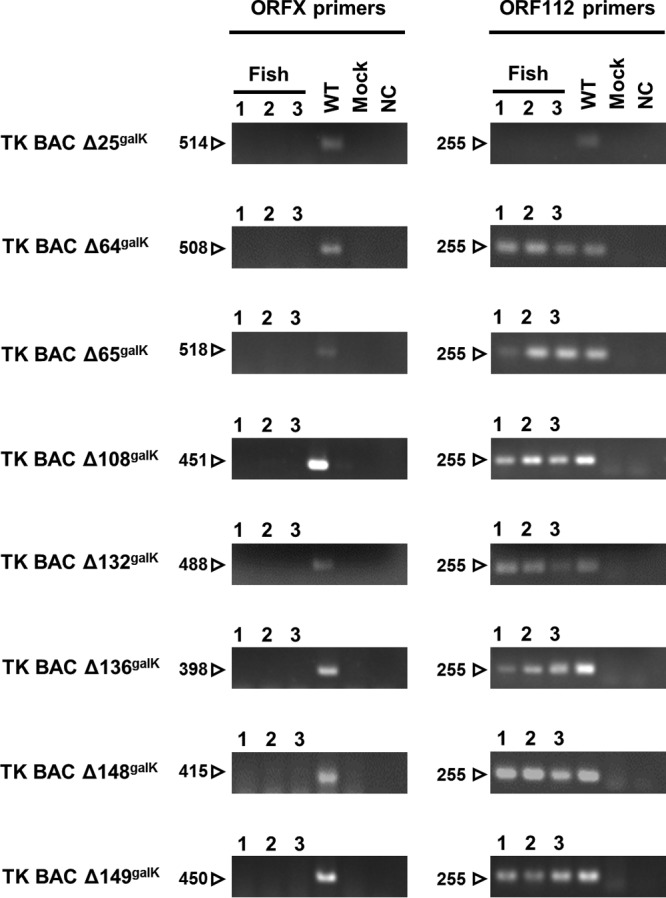
PCR detection and characterization of CyHV-3 genomes recovered from infected dead carp. DNA was extracted from the gills of three dead carp from each of the groups infected with the recombinant viruses deleted for a nonessential gene (TK BAC ΔORFX^galK^) during the course of the experiment described in the legend of Fig. 6. In the TK BAC Δ25^galK^ infections, no fish died, and hence three fish were selected randomly from among the living fish at 30 dpi. A carp infected with TK BAC virus and a mock-infected carp were used as positive and negative PCR controls, respectively. PCRs were performed with the appropriate ORFXintfw/ORFXintrev primer pair and with the ORF112intfw/ORF112intrev primer pair. The images are photographs of agarose gels. The numbers on the left of each panel are marker sizes (bp). NC, negative control.

To ensure that each group of fish was infected with the correct virus and to exclude any possibility of viruses spreading among the tanks, PCR assays were performed on three randomly selected dead fish from each infected group (for TK BAC Δ25^galK^-infected fish, three fish were selected randomly from the living fish at 30 dpi), one fish infected with the TK BAC virus, and one mock-infected fish selected randomly at the end of the experiment (Fig. 7). PCR performed with the ORF112intfw/ORF112intrev primer pair confirmed that samples from all but one group contained the CyHV-3 genome (the exception being the TK BAC Δ25^galK^-infected group). PCR performed with primers specific for the deleted ORFs confirmed that each group had been infected with the correct virus and that cross-contamination among tanks had not occurred (Fig. 7).

### Testing of an ORF25-deleted recombinant as an attenuated vaccine candidate against CyHV-3.

**FIG 8:**
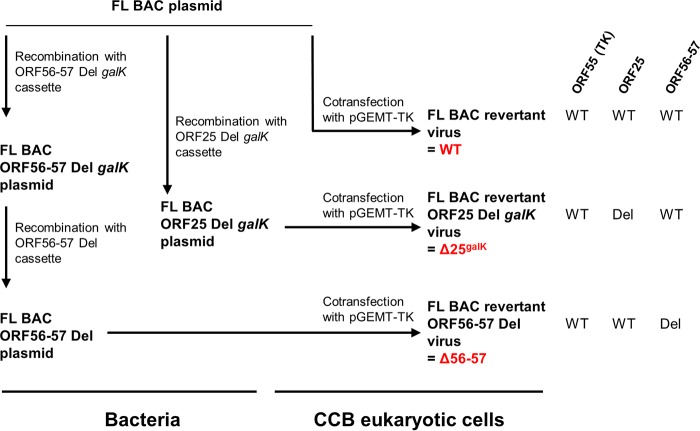
Flow chart of the production of CyHV-3 recombinants deleted for ORF25 or ORF56-57. Recombinants were given a short name (in red) for use in the text. Viral genotypes for ORF55 (TK), ORF25, and ORF56-57 are given on the right. Del, deleted; WT, wild type.

**FIG 9:**
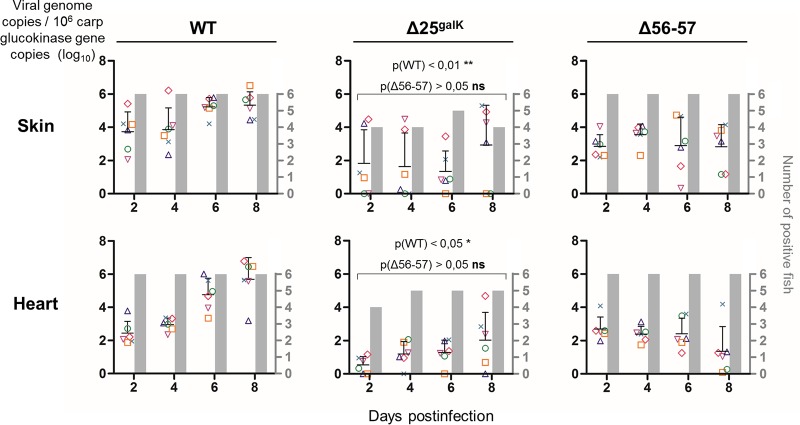
Effect of ORF25 deletion on viral tropism according to qPCR analysis. At time zero, carp (average weight, 6.10 g ± 1.39 g; average age, 6 months old) were infected for 2 h by immersion in water containing the virus indicated at 800 PFU/ml and then returned to larger tanks. At the indicated time points, fish were sampled and submitted to qPCR analysis. The data obtained for each fish (according to viral genotype and time postinfection) are represented by the same symbol to allow correlation of the data obtained for the two tested organs. The number of viral genome copies is expressed as the log_10_ per 10^6^ carp glucokinase gene copies. Individual values represent the mean of duplicate measurements. Mock-infected fish were used as a negative control and no viral genome copies were detected in these fish. The number of positive fish among six analyzed fish is represented by gray bars. Viral charges [viral genome copies/10^6^ carp glucokinase gene copies (log_10_)] were compared using one-way ANOVA taking the viral genotype as a variable.

The attenuation described above for TK BAC Δ25^galK^, from which ORF25 was deleted and encoding a truncated form of TK, suggested that a virus deleted only for ORF25 would have potential as an attenuated recombinant vaccine against CyHV-3. The requisite recombinant (Δ25^galK^, with ORF25 deleted and encoding a wild-type TK) was produced by following the procedure described in Fig. 8 and in Materials and Methods. The failure to detect virus by PCR in the gills of fish infected with the TK BAC Δ25^galK^ virus (Fig. 7) suggests that deletion of ORF25 could affect the ability of deleted virions to infect fish or to spread within their host. To test this hypothesis and to compare the tropism of a virus lacking ORF25 (Δ25^galK^) to the tropism of a previously described recombinant vaccine candidate (deleted for ORF56 and ORF57, Δ56-57), the following experiment was performed. Fish were infected with the FL BAC revertant virus (WT, wild-type for TK and for ORF25), FL BAC revertant ORF25 Del galK virus (Δ25^galK^, wild-type for TK but with ORF25 deleted), or the FL BAC revertant ORF56-57 Del virus (Δ56-57, wild-type for TK but with ORF56-57 deleted) (Fig. 9). The viral charge was measured by qPCR over time in the skin (the portal of entry of CyHV-3) and the heart (selected as an internal organ). The results demonstrated that the ORF25 deleted virus infects fish through skin infection and then spreads to internal organs as reported previously for the WT parental virus and the Δ56-57 virus (15). However, compared to the parental WT virus, the replication of the ORF25 deleted virus was reduced in intensity and duration to levels similar to those observed for the Δ56-57 virus.

**FIG 10:**
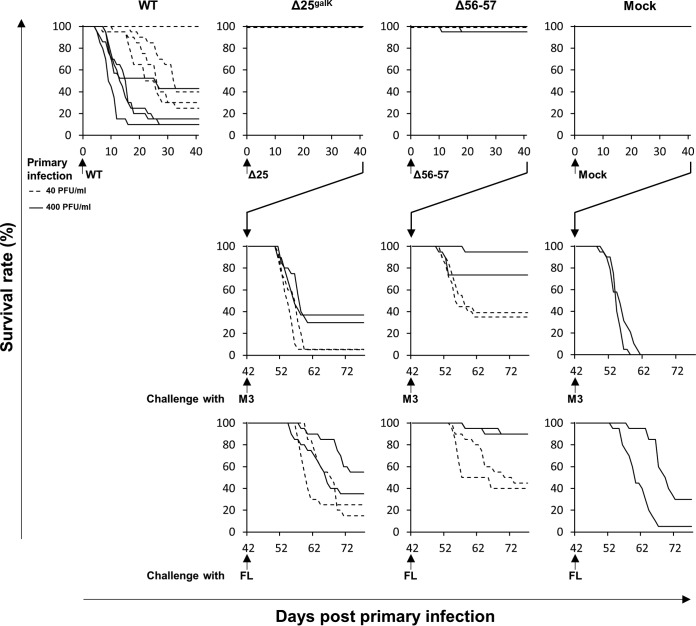
Testing of Δ25^galK^ and Δ56-57 deletion recombinants as attenuated recombinant vaccine candidates. The virulence of the recombinant viruses indicated was first tested in carp (first line of graph: four replicates per condition; *n* = 20 fish; average weight, 2.42 ± 0.95 g; average age, 6 months old). On day 0, fish were mock infected or infected for 2 h by immersion in water containing 40 (dotted line) or 400 (continuous line) PFU of virus/ml. The survival rate was measured over a period of 42 days. The immune protection conferred by primary infection was then tested for Δ25^galK^ and Δ56-57-infected fish at 42 dpi by distributing mock-infected fish and fish that survived the primary infection into duplicate tanks (*n* = 20) and challenging them by cohabitation with fish infected with the M3 strain (middle line of graphs) or the FL strain of CyHV-3 (lower line of graphs). The survival rate was measured according to time postchallenge.

Next, the virulence of the Δ25^galK^ virus was compared to that of the WT strain and the previously described Δ56-57 attenuated virus (Fig. 10) (15). A high degree of attenuation of Δ25^galK^ was indicated by the finding that infected fish exhibited no mortality or symptoms (Fig. 10). The level of immune protection conferred by primary infection with Δ25^galK^ or Δ56-57 virus was tested by challenging infected fish with the M3 or FL strains of CyHV-3 (Fig. 10, middle and lower lines of the graph, respectively). The fish vaccinated with Δ25^galK^ exhibited a poor level of protection, independent of the dose used for primary infection and the challenge strain used, with the relative percentage survival at a maximum of 34 when the fish were vaccinated at a dose of 400 PFU/ml and challenged with the M3 strain. In comparison, vaccination with the Δ56-57 attenuated virus resulted in a relative percentage survival of 84 under the same conditions (Fig. 10).

The data above demonstrated that the VTP encoded by ORF25 is nonessential for viral replication *in vitro* but acts as a key virulence factor *in vivo*. ORF25 is the prototypical gene of the ORF25 family, which contains six paralogs (ORF25, ORF26, ORF27, ORF65, ORF148, and ORF149) encoding type I transmembrane proteins (5). Although ORF26 is a pseudogene, all other paralogs encode CyHV-3 VTPs. The FL strain used in this study as the parental strain encodes a disrupted ORF27, as has been reported for other strains of CyHV-3 (5). Individual deletions of each member of the ORF25 family in this ORF27-negative background revealed that none of them is essential for viral replication *in vitro*, although it is possible that this is the consequence of redundant functions expressed by the paralogs. The attenuation observed for a mutant lacking ORF25 suggested that this locus might serve as a target for generating recombinant attenuated vaccines. However, there are two points of caution in relation to this hypothesis: the ORF25 deletion virus induced weak immune protection, and it is possible that reversion to virulence could occur by mutation of the paralogous genes.

### Concluding remarks.

Herpesvirus VTPs play crucial roles in the viral life cycle. Although the functions of VTPs in members of the family Herpesviridae have been studied extensively, very little information is available on VTPs in members of the family Alloherpesviridae. The very distant evolutionary origin of these families has resulted in a lack of detectable genetic similarity between VTPs in the two groups. This situation makes it impossible to advance hypotheses on the roles of alloherpesvirus VTPs on the basis of the much more extensive knowledge available for other herpesviruses. The present study of the archetypal fish alloherpesvirus has enabled major steps to be taken toward characterizing alloherpesvirus VTPs: (i) the number of CyHV-3 VTPs was expanded from 14 to 16; (ii) these were characterized as consisting of eight essential and nine nonessential proteins; (iii) individual deletion of seven nonessential VTPs affected viral growth *in vitro*, and individual deletion of two affected virulence *in vivo*; and (iv) a mutant lacking ORF25 proved to be highly attenuated *in vivo* but induced a poor immune protection against a lethal challenge. This study provides a firm basis for the further study of alloherpesvirus VTPs.

## MATERIALS AND METHODS

### Cells and viruses.

Cyprinus carpio brain (CCB) cells (16) were cultured as described previously (14). The CyHV-3 FL strain was isolated in Belgium from a fish that had died from CyHV-3 infection and had been used to produce the FL BAC plasmid (14). Other CyHV-3 strains from different geographical origins were also used: the M3 strain from Belgium, the U strain from the United States (5); the J strain from Japan (5), the E strain from England (kindly provided by K. Way), the T strain from Taiwan (17), the GZ11 strain from China (18), and the KHV-I and I strains from Israel (5). The attenuated Cavoy strain from Israel was produced by passaging in cell culture (19).

### Production and purification of CyHV-3 virions.

CCB cells were infected with the CyHV-3 FL strain at a multiplicity of infection (MOI) of 0.02 PFU/cell (20). The supernatant was harvested at the beginning of viral release, when cell lysis was minimal (4 dpi). Virions were purified from the cell supernatant and monitored for purity as described previously (9).

### SDS-PAGE and LC-MS/MS proteomic analysis.

Virion proteins were separated by SDS-PAGE and detected by Coomassie blue staining. The entire lanes of the gel were cut into 30 equally sized slices, which were subjected to in-gel trypsin digestion (21). Peptides were separated by reversed-phase chromatography using a 40-min CAN gradient (4 to 45%) and analyzed by using an HCT ultra-ion trap (Bruker) in data-dependent AutoMS (2) mode with a scan range of 100 to 2,800 *m/z* and three averages, and five precursor ions at 300 to 1,500 *m/z* were selected from the MS scan. Precursors were actively excluded within a 0.5 min window, and all singly charged ions were excluded. The MS data were processed by using Mascot distiller, and all single-slice analyses were merged into a single data set. The data were searched for matches to CyHV-3 protein sequences (160 entries) by using an in-house Mascot 2.2 server (Matrix Science). The default search parameters used were as follows: enzyme = trypsin, maximum missed cleavages = 1, fixed modifications = carbamidomethyl (C), variable modifications = oxidation (M), peptide tolerance ± 1.3 Da; MS/MS tolerance = ± 0.5 Da, and instrument = ESI-TRAP. Only sequences identified by a Mascot score of >30 were considered, and single peptide identification was systematically evaluated manually. In addition, the emPAI (22) value was calculated to estimate relative protein abundance in the GC extract only.

### Antibodies.

Mouse pAbs against CyHV-3 ORF148 or ORF149 were produced by customized DNA immunization (DelphiGenetics).

### Cloning and site-directed mutagenesis of CyHV-3 ORFs.

DNA fragments containing CyHV-3 ORFs of interest plus at least 200 bp upstream and downstream were amplified by PCR using CyHV-3 FL BAC DNA as a template (see Table 3 for primer details). The amplification products were TA cloned into the pGEM-T-Easy vector (Promega), and the resulting clones (pGEM-T-ORFX WT, X standing for the number of the ORF tested) with the correct sequence were used as a recombination cassette or as a template for mutagenesis. For ORFs encoding VTPs, site-directed mutagenesis was used to create 1- or 2-bp substitutions changing a tyrosine or a tryptophan codon into a stop codon, resulting in pGEM-T-ORFX NS. Oligonucleotide primers were designed by using the QuikChange primer design tool (http://www.genomics.agilent.com/primerDesignProgram.jsp) (Table 3).

**TABLE 3:** Oligonucleotide primers^*a*^

Analysis	Primer	Sequence (5′–3′)^*b*^	Coordinates or accession no.
Synthesis of inserts			
CyHV-3 ORF25	ORF25outF	CCTCCGACTCTGAAGACGAT	45389–45408
	ORF25outR	GGTACGTGATGCTGTAGAAG	47569–47588
CyHV-3 ORF32	ORF32outF	CCAGGTCCAGGCAGTCTC	54216–54233
	ORF32outR	ATGCGCGTCTACAAGATCG	55619–55601
CyHV-3 ORF59	ORF59outF	GACGCCTGTCTCTGGTAGA	101541–101559
	ORF59outR	GCGTCTCCAAAAAGAGCGG	102941–102923
CyHV-3 ORF64	ORF64outF	GTCGAGATATCCGAGGCAGA	119716–119735
	ORF64outR	TTCATAGCCACAACCGGAGT	122203–122184
CyHV-3 ORF65	ORF65outF	TGGAGAACATCAAGCAGCAC	121920–121939
	ORF65outR	GAAGTCTGAAACTGTCTGAATG	124106–124127
CyHV-3 ORF81	ORF81outF	GGTTTAGTCCAAATCCGACCT	150519–150539
	ORF81outR	GCAGCTTCTGCTTCAGAGTG	152023–152004
CyHV-3 ORF83	ORF83outF	ACACGCTGATGGTCACGAG	152549–152567
	ORF83outR	ACACCAACCAGTTCGTGCAG	153638–153619
CyHV-3 ORF99	ORF99intF	GCTTAGCCTGTTCGGCAC	184445–184462
	ORF99intR	AAGATCTGGGACACGGACTG	185515–185496
CyHV-3 ORF106	ORF106outF	GCTGACACCTGTCACAACCA	195629–195648
	ORF106outR	TCGTGAGGACAAACCGTCTC	196189–196170
CyHV-3 ORF108	ORF108outF	GATGATGAAGGGTGTTCATG	201067–201086
	ORF108outR	CATCTACAAGTCGGACAACC	201937–201956
CyHV-3 ORF115	ORF115outF	CACGTAAACGAAGCCCCATA	207951–207970
	ORF115outR	ATTCGTGGCGGCTGTTATAC	210734–210715
CyHV-3 ORF131	ORF131outF	GCCCTGGTCCTCGTACTTTT	224971–224990
	ORF131outR	CTGATCAGATTCTCAGGAGCAG	227271–227250
CyHV-3 ORF132	ORF132outF	GCTCCTGAGAATCTGATCAG	227252–227271
	ORF132outR	GTTAATGGTCACAGAAGCGC	228155–228174
CyHV-3 ORF136	ORF136outF	GTGTCAAGTACGTGGAGCGT	231157–231176
	ORF136outR	GGCCTCTGAATGTGTTATTGC	231991–232011
CyHV-3 ORF148	ORF148outF	CATGGTTCGAGGTTGTGAAG	253808–253827
	ORF148outR	CAGCATTCGTCTCTTAGTGC	255933–255952
CyHV-3 ORF149	ORF149outF	ACCCTATCATGATTGACGGC	255770–255789
	ORF149outR	CTTTCGTTCTACTGTTCCTC	258172–258191
Synthesis of *galK* recombination cassettes			
ORF 25 Del *galK*	ORF25 *galK* F	GTCATTTTTCCTCGTGGTGTGCAGCTTTTTCTAACGTGTGGTAGGACGCTAACCATTACCAAGTAAACCATTACG*CCTGTTGACAATTAATCATCGGCA*	45495–45569
	ORF25 *galK* R	TGACCAGGGTAGAATGATAGACCCGCCGCTAAGAAAAATGACTCTCCGTCCGCTACCGTCGCCCGCTATAGTGTT*TCAGCACTGTCCTGCTCCTT*	47376–47450
ORF 32 Del *galK*	ORF32 *galK* F	ATAATACGCACCCTGGTCACTTTCTTTAACGTGTCCGTGTCGGGCTCCCTACACGTTTCCTCCGAGTCGCCGATC*CCTGTTGACAATTAATCATCGGCA*	54417–54491
	ORF32 g*alK* R	CTGTATCCCATCAGGGGCTGATCTGCTGACGGCCTCGACGTGGTGGTGATGGTGGTGGTGGTGGTGATGATGATG*TCAGCACTGTCCTGCTCCTT*	54491–54417
ORF 59 Del *galK*	ORF59 *galK* F	CCCGCACGACGTCGGACGCTCCCTACCGAGTGAGCCGTCTCTCTGACCCGGGTCGTGCGGGCGCCACCAGCCCTT*CCTGTTGACAATTAATCATCGGCA*	101961–102035
	ORF59 *galK* R	AGCAGTGGTAGTCCTCTCACCCCCCCCCCCCCCCCCTTGACCTTCACCCTTCACCCCTAACCCCAACAACCAACC*TCAGCACTGTCCTGCTCCTT*	102521–102447
ORF 64 Del *galK*	ORF64 *galK* F	TGGTCCTCCCATGCTTTTTATTCAATCCTGCTCCTCTTGAAAAGCTTAAG*CCTGTTGACAATTAATCATCGGCA*	119814–119863
	ORF64 *galK* R	GTACACTATATTGCGTTTATTTGTTTTTCATAAAAAGCACATACATAATA*TCAGCACTGTCCTGCTCCTT*	122082–122033
ORF 65 Del *galK*	ORF65 *galK* F	CAATATAGTGTACACCGTGGTCTTTATTAATTTGATAACATAATAATGTA*CCTGTTGACAATTAATCATCGGCA*	122070–122119
	ORF65 *galK* R	CAAGACGCCTCACACGACGGAAACCCGCAACACCACCCCACAAACAAACG*TCAGCACTGTCCTGCTCCTT*	123911–123960
ORF 81 Del *galK*	ORF81 *galK* F	GCACTTGAGAGCAGCGGAGGAGAAGACCGGTGCATCCATCTCGACGCTCGTCAGGTTGTGACTGTCCTCGTCCTC*CCTGTTGACAATTAATCATCGGCA*	150722–150796
	ORF81 *galK* R	CGGCTGCTGTGTTTTGTGGTGCTGGCCTCGACTGTGGGGTGGTGGTGGCGGCGACTGCGAGAGGCTTCGGAA*TCAGCACTGTCCTGCTCCTT*	150796–150722
ORF 83 Del *galK*	ORF83 *galK* F	CACAATAAAAAGCTCCCCTCGGGGATCACCAAGACAACCTTTCAGTAGCC*CCTGTTGACAATTAATCATCGGCA*	153481–153530
	ORF83 *galK* R	TCTTCGCCCTCGCCCTCGTCCCTCAACCTGTTGTACCTATACGCGCTCAT*TCAGCACTGTCCTGCTCCTT*	152758–152807
ORF 99 Del *galK*	ORF99 *galK* F	TGCAGGCCACCGCCGCCAACGCCGAGATGTCCAACAACCTCCAGACGCAG*CCTGTTGACAATTAATCATCGGCA*	184714–184763
	ORF99 *galK* R	AGGGCTTGCTCTTGAGGAAGGGCTGGTTGACGGGGCAGCCCTGCAGGTAA*TCAGCACTGTCCTGCTCCTT*	185214–185165
ORF 106 Del *galK*	ORF106 *galK* F	ACTACTCGCCCACCGCCTTATACAACTTCACCGCGTATCCGCCGTACATC*CCTGTTGACAATTAATCATCGGCA*	195727–195776
	ORF106 *galK* R	GGGTTTGGTAGAGGGCGCTGCGGGGGATGAGCCCGACGATACCGCTGCGG*TCAGCACTGTCCTGCTCCTT*	196063–196014
ORF 108 Del *galK*	ORF108 *galK* F	CACTTAGTCTACCACTCACTTTACCTTACAACCACCCAGACACCAACACACCACACCACCCACACTACGAGCGAG*CCTGTTGACAATTAATCATCGGCA*	201156–201230
	ORF108 *galK* R	ACCAAGCAACACGTTCATGTGATTTTTTTTTATTGTTGATGTTGTTTGATGTATTACAGTTACAATAAACGTAAT*TCAGCACTGTCCTGCTCCTT*	201819–201893
ORF 115 Del *galK*	ORF115 *galK* F	ATCGTGTCCTCCGTCTCGGTCATCTCTGTGGTTTTTTCCAGCTTTGCGCC*CCTGTTGACAATTAATCATCGGCA*	208152–208226
	ORF115 *galK* R	GCTCAAAATAAAAAAAACATGTCTGGACCTTTACATCTTGTTTCAATAAT*TCAGCACTGTCCTGCTCCTT*	210641–210567
ORF 131 Del *galK*	ORF131 *galK* F	GTGAGGGAGTGATATGGAGTGAACGTAAATGGAGGGGCGCTGCGGAGGTT*CCTGTTGACAATTAATCATCGGCA*	225410–225484
	ORf131 *galK* R	TCGAGACGCCCGAACTGGTCGAGGCCTACGTGAACGACGTCAAGGTCCGC*TCAGCACTGTCCTGCTCCTT*	226426–226352
ORF 132 Del *galK*	ORF132 *galK* F	TCAAGTCTTTATTATTACTTTATTTAATGGGGTTCGGTTGGAGAAGAGCAGTCAAGTGAGCAGAAGAGAAGATCG*CCTGTTGACAATTAATCATCGGCA*	227369–227443
	ORF132 *galK* R	AACTGAGGCCTCTCCGGCTATCCGCACCGAGTGATATACCGCCCGCCACTACCCCAGAACCCCCACACAGCCGCA*TCAGCACTGTCCTGCTCCTT*	227957–228031
ORF 136 Del *galK*	ORF136 *galK* F	AAGCTTGCCTTTGAAACATGGAAAAGAGCTCTCGTGTGTTTCTTTAGAATAAGACGACGTCGAGCGACGACCATC*CCTGTTGACAATTAATCATCGGCA*	231264–231337
	ORF136 *galK* R	TGTTGGTTTGCTTTGGCTTGATAGCACACAAAAATCACATTTGTAAGAGTTTTATTGTTTGACAATGATACATCA*TCAGCACTGTCCTGCTCCTT*	231801–231875
ORF 148 Del *galK*	ORF148 *galK* F	GAGGGTAGAGAGGGCTGGAGAGTAGGAGAGGAGAGAGGGGAAAGTTTTGAAGTTCTTGCACATGTTGAGGGGAAG*CCTGTTGACAATTAATCATCGGCA*	253881–253955
	ORF148 *galK* R	AAAACCAAAATTTTTCTGTTATCGTATCGTGATGAGCGCGACCGAGGCGACGGTCCAGTAACTCCGCCGTCAATC*TCAGCACTGTCCTGCTCCTT*	255780–255854
ORF 149 Del *galK*	ORF149 *galK* F	TTTGCTTTTTTTATTATGTGATGCTGTAGTGTACACACGTATTAGAAACAATAGTAGAGCAAGCAAGCAAAAGC*CCTGTTGACAATTAATCATCGGCA*	255861–255935
	ORF149 *galK* R	TTTTCTTTTGACGCGCAATGACCGACGGCTCAAGTGACGGCTACCTAGACGATACGTCACAGACGGCTGAGGACC*TCAGCACTGTCCTGCTCCTT*	257997–258071
Nonsense mutagenesis of essential genes^c^			
ORF32	ORF32.T237G-sense	GACAACGGCTGGGGCTA*G*ACCTTTCTGATCCAATC	54711–54745
	ORF32.A237C-antisense	GATTGGATCAGAAAGGT*C*TAGCCCCAGCCGTTGTC	54745–54711
ORF59	ORF59.C144G-sense	CATGCAGCGCTA*G*CAACTGTGCGAG	102315–102291
	ORF59.G144C-antisense	CTCGCACAGTTG*C*TAGCGCTGCATG	102291–102315
ORF81	ORF81.C207G-sense	TTGCGCACGCCATGTA*G*TCCAACGATCCC	150987–151015
	ORF81.G207C-antisense	GGGATCGTTGGA*C*TACATGGCGTGCGCAA	151015–150987
ORF83	ORF83.G225A-sense	GTTCATGTACTG*A*GTCACCCTACGC	153268–153244
	ORF83.C225T-antisense	GCGTAGGGTGAC*T*CAGTACATGAAC	153244–153268
ORF99	ORF99.C3039G-sense	CAACGACAGGTTCTCGTA*G*GTGGCGAGCC	184946–184974
	ORF99.G3039C-antisense	GGCTCGCCAC*C*TACGAGAACCTGTCGTTG	184974–184946
ORF106	ORF106.CA108AT-sense	CTTCGTCGTCTA*AT*AGGCCCAGCAC	195872–195896
	ORF106GT108TA-antisense	GTGCTGGGCCT*AT*TAGACGACGAAG	195896–195872
ORF115	ORF115.C744A-sense	GGACCTGGTCCTGTA*A*TCCCAGACGAACGTG	208955–208985
	ORF115.G744T-antisense	CACGTTCGTCTGGGA*T*TACAGGACCAGGTCC	208985–208955
ORF131	ORF131.C735G-sense	TCCAGGGCCGTGTA*G*GAGGCCGC	226054–226032
	SDM131.G735C-antisense	GCGGCCTC*C*TACACGGCCCTGGA	226032–226054
Synthesis of probes for Southern analysis (probe name)			
*GalK*	*GalK*.Int2-F	AGGTGAGGAACTAAACCCAG	
	*GalK*.Int2-R	CGTATTGCAGCAGCTTTATC	
ORF32	ORF32.SB-F	CAGATGTGGCTGGACATGA	54567–54585
	ORF32.SB-R	GCTACCCAGGTGGTGTTGTT	55006–54987
ORF59	ORF59.SB-F	GTTCTGCGTCGAGGATGATG	102042–102061
	ORF59.SB-R	CGTTCAGCACCTCGGTCTAC	102427–102408
ORF81	ORF81.SB-F	AAAGCTCAACTGGCCAAGAG	150809–150828
	ORF81.SB-R	ACACCGCCGTCTTCGAGTAG	151259–151240
ORF83	ORF83.SB-F	TGCTCTCCACGTCCTTGTA	152838–152856
	ORF83.SB-R	ATGTCTCCTTTGTGCGGTC	153480–153462
ORF99	ORF99.SB-F	CCTCCAGACGCAGATCAACT	184751–184770
	ORF99.SB-R	CTTGAGGAAGGGCTGGTTG	185204–185186
ORF106	ORF106.SB-F	GCAGCAGCAGCAGAAGTG	195783–195800
	ORF106.SB-R	TCAGTTGGTCTTGGGGCC	196013–195996
ORF115	ORF115.SB-F	CTACGCCAACGACGAACC	209426–209443
	ORF115.SB-R	AGCACCACGAACCACACTC	209776–209758
ORF131	ORF131.SB-F	ACTGGAGCGGGTGATAGTTG	225487–225506
	ORF131.SB-R	GGACTCGTCGTGCCTCTC	225937–225920
PCR			
ORF25	ORF25int_F	ATCAAGCGCTACGACGACTT	45948–45967
	ORF25int_R	TGTTGCAGGAGGTGTAGACG	46442–46461
ORF64	ORF64int_F	CCATAGTCCAGGACGACGAT	121431–121450
	ORF64int_R	TGCTGCTTGATGTTCTCCAC	121919–121938
ORF65	ORF65int_F	GATGGTCATGTTGGTGTTGC	122996–123015
	ORF65int_R	CCAAGAACGAGCTCTTCACC	123494–123513
ORF108	ORF108int_F	ACCAACTACACGACCGTCTC	201246–201265
	ORF108int_R	CCGGGTTGGTGTAGGTAGAA	201677–201696
ORF132	ORF132int_F	TTGGTTTTTGTTGGTGACGA	227468–227487
	ORF132int_R	TGACGGGTTCCAAGATTAGC	227936–227955
ORF136	ORF136int_F	CTGGTTACATGGGTGGCTTT	231360–231379
	ORF136int_R	TAGCCCCTGTTGTAGGATGC	231738–231757
ORF148	Pr148fw2	GGTTGTTGGAGTAGTGGTGC	254196–254215
	Pr148rev2	CTCATTCAGGCTCGGAGAC	254592–254610
ORF149	Pr149fw1	GTAGTCGCTGGATGTGACG	256662–256680
	Pr149rev1	GTCAACACGGACTGCTCCG	257093–257111
ORF112	ORF112intFW	CCAGTGTCATCACCACAAGC	205336–205355
	ORF112intREV	ACGGACATCCTGGGTATCAA	205571–205590
qPCR			
CyHV-3 ORF89	KHV-86F	GACGCCGGAGACCTTGTG	AF411803
	KHV-163R	CGGGTTCTTATTTTTGTCCTTGTT	
	KHV-109P	(6FAM) CTTCCTCTGCTCGGCGAGCACG (BHQ1)	
Carp glucokinase	CgGluc-162F	ACTGCGAGTGGAGACACATGAT	AF053332
	CgGluc-230R	TCAGGTGTGGAGCGGACAT	
	CgGluc-185P	(6FAM) AAGCCAGTGTCAAAATGCTGCCCACT (BHQ1)	

a Coordinates are listed in relation to GenBank accession number NC_009127.1.

b Underlined: segments correspond to the CyHV-3 sequence; italicization indicates sequences corresponding to *galK* sequence. Mutated nucleotides are both underlined and italicized.

### Production of CyHV-3 FL BAC recombinant plasmids and CyHV-3 recombinant viruses using BAC cloning and prokaryotic recombination technologies.

CyHV-3 FL BAC recombinant plasmids and CyHV-3 recombinant viruses were produced on the basis of the strategy outlined in Fig. 2 and 8, by using a two-step galactokinase gene (*galK*) positive/negative selection in bacteria (15, 23). Single-gene deleted recombinant plasmids were produced for various ORFs by replacing the sequence (initiation codon to stop codon) by a *galK* expression cassette (*galK* positive selection). Recombination cassettes encoding *galK* were produced by PCR using the primers listed in Table 3 and the p*galK* vector as the template. The derived ORFX Del *galK* cassette consisted of the *galK* gene flanked by 75-bp sequences homologous to the regions of the CyHV-3 genome immediately upstream and downstream of ORFX. The flanking sequences served as targets to induce homologous recombination with the FL BAC parental plasmid in competent E. coli cells, resulting in replacement of the entire ORF by the *galK* expression cassette (FL BAC ORFX Del *galK* plasmid).

The recombinant FL BAC plasmids were transfected into permissive CCB cells in the presence of polyethylenimine (PEI). Transfection of the BAC plasmids and eventual reconstitution of infectious virus were monitored by epifluorescence microscopy, since the FL BAC plasmids and derived viruses contained an EGFP expression cassette. For mutations that did not prevent reconstitution of infectious virus (i.e., in nonessential genes), cell supernatants were collected and the viruses were amplified, leading to FL BAC recovered ORFX Del *galK* viruses. For mutations that prevented reconstitution of infectious virus (i.e., in essential genes), further investigations were performed. For each ORF identified as being essential for viral growth, WT revertant (Rev1) and nonsense (NS) mutation clones were produced by using a second recombination process (*galK* negative selection). ORFX Rev1 and ORFX NS recombination cassettes were produced by PCR using WT FL BAC DNA and pGEMT-ORFX NS as the templates, respectively (see Table 3 for primers). The resulting FL BAC ORFX Rev1 and FL BAC ORF X NS plasmids were transfected into CCB cells as described above. To exclude the possibility that the lack of infectivity of the NS-null recombinant BAC plasmids was due to unexpected mutations during BAC manipulation, FL BAC ORFX NS plasmids were also transfected into CCB cells together with a WT DNA fragment containing the appropriate ORF and upstream and downstream flanking regions, in order to induce reversion to a WT sequence after homologous recombination in eukaryotic cells.

### Genetic characterization of CyHV-3 recombinant BAC plasmids and derived recombinant viruses.

CyHV-3 FL BAC recombinant plasmids and derived recombinant viruses were characterized by sequencing regions of interest and by assessing the sizes of SacI fragments by agarose gel electrophoresis and Southern blotting (24). Probes for Southern blotting were produced by PCR using the primers listed in Table 3. CyHV-3 recombinant viruses deleted for nonessential genes (the FL BAC recovered ORFX Del *galK* viruses and the FL BAC revertant ORF25 Del *galK* virus) were characterized further by full-length genome sequencing as described previously (15).

### Indirect immunofluorescence staining.

Cells grown on glass coverslips were fixed in phosphate-buffered saline (PBS) containing 4% (wt/vol) paraformaldehyde at 4°C for 15 min and then 20°C for 10 min. After a washing step with PBS, samples were permeabilized in PBS containing 0.1% (vol/vol) NP-40 at 37°C for 15 min (15). Immunofluorescence staining (incubation and washes) was performed in PBS containing 10% (vol/vol) fetal calf serum (FCS). Monospecific mouse pAbs (dilution 1:100) against CyHV-3 ORF148 or ORF149 were used as the primary antibody, followed by Alexa Fluor 568 goat anti-mouse immunoglobulin G(H+L) (Invitrogen) as the secondary antibody (1:1,000 dilution). After a washing step, the cells were mounted by using Prolong Gold antifade reagent with DAPI (4′,6′-diamidino-2-phenylindole; Invitrogen). Samples were analyzed by confocal microscopy using a Leica SP5 confocal microscope (14).

### Multistep growth curves.

Triplicate cultures of CCB cells were infected with CyHV-3 viruses at an MOI of 0.05 PFU/cell. After incubation for 2 h, the cells were washed with PBS and overlaid with Dulbecco modified Eagle medium (DMEM) containing 4.5 g/liter glucose and 10% (vol/vol) FCS. The supernatant was removed from the infected cultures at successive intervals (2, 4, and 6 dpi) and stored at −80°C. Titers of infectious viral particles were determined by duplicate plaque assays in CCB cells (24).

### Plaque size assay.

CCB cells grown in 6-well plates were infected with viruses at an MOI of 200 PFU/well. After incubation for 2 h, the cells were overlaid with DMEM containing 10% (vol/vol) FCS and 0.6% (wt/vol) carboxymethyl cellulose (medium viscosity; Sigma) in order to obtain isolated plaques (25). At successive intervals after infection, the plaques were fixed in PBS containing 4% (wt/vol) paraformaldehyde at 4°C for 15 min and then 20°C for 10 min. Images were captured with a confocal microscope (Nikon A1R). For each virus, 25 plaques were measured in duplicate wells. The plaque area was measured by using ImageJ 1.46 software (26).

### Fish.

Common carp (Cyprinus carpio) were kept in 60-liter tanks at 24°C. Water parameters were checked twice per week. The health of the fish was confirmed by microbiological, parasitic, and clinical examination immediately prior to the experiments, all of which were preceded by an acclimation period of at least 2 weeks.

### Inoculation of fish with CyHV-3.

Two modes of inoculation were used: immersion in infectious water and cohabitation with newly infected fish. For inoculation by immersion, the fish were immersed for 2 h in constantly aerated water containing virus, the volume of water being adjusted to a biomass of around 10% on the basis of fish size and number. At the end of the incubation period, the fish were returned to 60-liter tanks. For inoculation by cohabitation, naive fish were immersed for 2 h in water containing 400 PFU/ml of the FL or M3 strain of CyHV-3. The newly infected fish were then released into tanks at a ratio of 2 newly infected fish per 20 fish to be infected.

### Ethics statement.

The maintenance and care of fish and the experiments conducted complied with the guidelines of the *European Convention for the Protection of Vertebrate Animals Used for Experimental and other Scientific Purposes* (CETS 123). The animal studies were approved by the local ethics committee of the University of Liège, Belgium (laboratory accreditation 1610008, protocol 1414). All efforts were made to minimize suffering.

### Quantification of virus genome copies in organs by real-time TaqMan PCR.

The virus genome was quantified by real-time TaqMan PCR as described previously (24), by amplifying fragments of the CyHV-3 ORF89 and carp glucokinase genes. The primers and probes are listed in Table 3.

### Statistical analysis.

Viral growth (Fig. 5A) and plaque size (Fig. 5B) results were compared by using two-way analysis of variance (ANOVA) with interactions, followed by a post hoc Student *t* test. qPCR results (Fig. 9) were compared using one-way ANOVA, followed by a post hoc Student *t* test. Survival curves (Fig. 6 and 10) were compared by using log-rank tests. These analyses were done by using GraphPad Prism 5. The variables used and comparisons retained for statistical illustrations are presented in the figure legends. Statistical significance is represented in the figures by asterisks as follows: *, *P* < 0.05; **, *P* < 0.01; and ***, *P* < 0.001.
